# Supplementary material for: Dissecting the Molecular Regulation of Natural Variation in Growth and Senescence of Two Eutrema salsugineum Ecotypes
Source: Int J Mol Sci. 2022 May 30;23(11):6124. doi: 10.3390/ijms23116124 (PMC9181637; doi:10.3390/ijms23116124)
Supplement: Supplementary file 1 [file ijms-23-06124-s001.zip › Supplementary method.pdf]

Supplementary method. A hydroponic culture system for growing *Eutrema salsugineum*

Plant material

E. salsugineum (Shandong and Xinjiang ecotype)

Equipment and chemicals

72-well 0.5 mL centrifuge tube box with cover (Sangon, Shanghai, China)

1.5 mL graduated centrifuge tube (Sangon, Shanghai, China)

Scalpel or sharp knife

10 × Hoagland solution

Murashige and Skoog solid medium

Agarose I-M (BBI, Canada)

Clean bench

Seeds sterilization and germination on MS solid medium

Approximately 100 seeds per culture box were surface-sterilized in 1.5 mL centrifuge tubes with 0.1% (v/v) Tween 80 in sodium hypochlorite solution (0.7% available chlorine) for 10 min. Seeds were then washed six times in sterile distilled water and sown on MS solid medium (MS salt + 3% (m/v) sucrose + 0.7% (m/v) agar, pH 5.8), stored in dark for 7 d at 4°C for stratification. After stratification, MS solid medium plates were transferred into chamber (16 h/8 h light-dark regime, 20 ± 2°C).

Agarose-Hoagland supporting medium

Prepare 1 × Hoagland solution (pH 5.8) with 5.5 g/L agarose and autoclave at 120 °C for 15 min.

Preparing culture vessels

Cut a 1.5 mL graduated centrifuge tube with a sharp knife (**Supplementary Figure 1A**). The first cut at the 0.1 mL mark and the second cut at the 0.5 mL mark and collected the mid-cone section (**Supplementary Figure 1B**). 1.5 mL fractionation centrifuge tubes and 72-well 0.5 mL centrifuge tube boxes (lids) (**Supplementary Figure 1C**) were autoclaved at 120°C for 15 minutes. The hydroponic box is shown in **Supplementary Figure 1D**. The following steps are performed under sterile conditions: Place the central conical section firmly on a 9 cm plate with the large diameter end facing up. Pour the melted agarose Hoagland medium into plates (about 3 mm deep) to plug the bottom of the cone. After the agarose has solidified in the plate, fill the pan with agarose Hoagland medium using a 1000 µl Gilson pipette. After the agarose has solidified in the cone, use forceps to place the cone into a 0.5 mL 72-well tube box. Four-day-old seedlings on MS medium were placed on cone surfaces filled with agarose. Cover the box with the lid, then transfer the boxes into chamber (16 h/8 h light-dark regime, 20 ± 2°C).

Plantlet growth in chamber

Open the box after two days. The box was filled with DDW each day and after 4 days the DDW was replaced with 1 × Hoagland solution. Fill the box with 1 × Hoagland solution each day and refresh the Hoagland solution every three days.

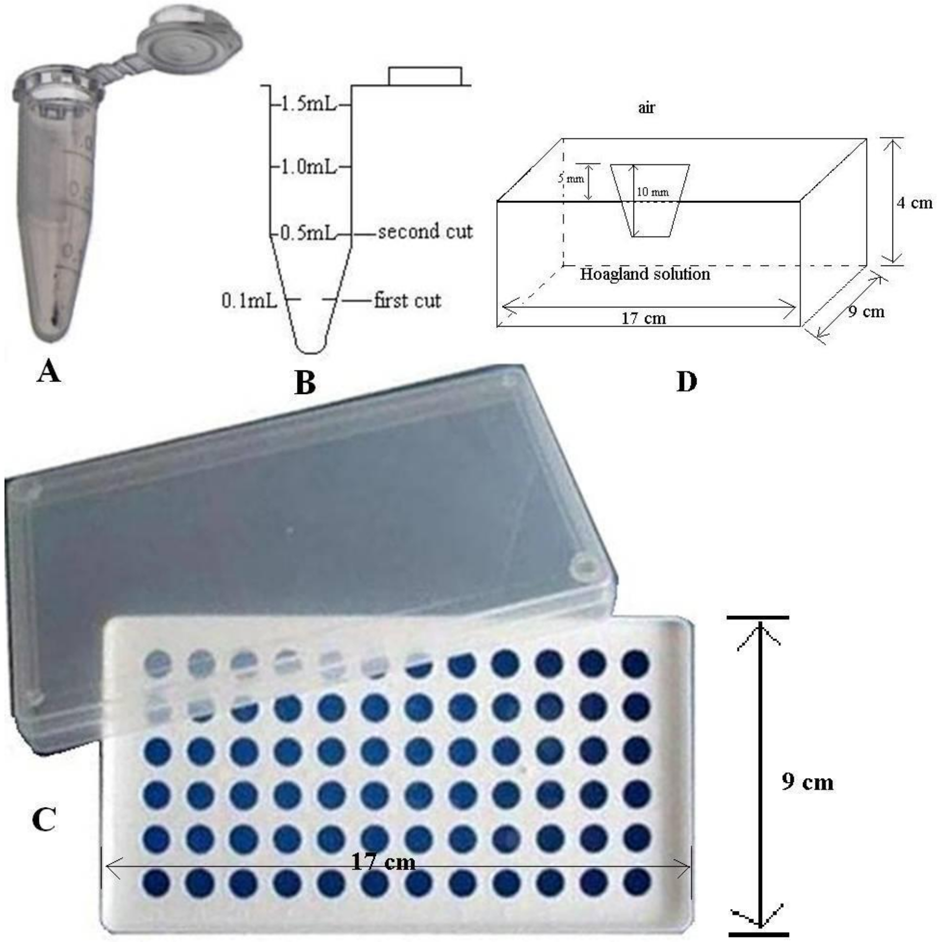

Figure S1. The system of hydroponic culture. **(A)** The 1.5 mL graduated centrifuge tubes. **(B)** Cutting positions for 1.5 mL centrifuge tubes. **(C)** 72-well 0.5 mL centrifuge tube boxes with cover. The 0.5 mL 72-well centrifuge tubes with caps. **(D)** The hydroponic culture box.
